# Adaptor protein LNK promotes anaplastic thyroid carcinoma cell growth via 14-3-3 ε/γ binding

**DOI:** 10.1186/s12935-019-1090-9

**Published:** 2020-01-09

**Authors:** Zhao-Ming Zhong, Xue Chen, Xiao Qi, Xue-Min Wang, Chun-Yan Li, Ru-Jia Qin, Shi-Qi Wang, Jin Liang, Mu-Sheng Zeng, Chuan-Zheng Sun

**Affiliations:** 1 Department of Head and Neck Surgery Section II, The Third Affiliated Hospital of Kunming Medical University/Yunnan Cancer Hospital, 519 Kunzhou Road, Kunming, China; 2 Department of Medical Oncology, The First Affiliated Hospital of Kunming Medical University, 295 Xichang Road, Kunming, China; 3 State Key Laboratory of Oncology in South China, Sun Yat-sen University Cancer Center, 651 Dongfeng Road East, Guangzhou, China

**Keywords:** Adaptor protein, Anaplastic thyroid carcinoma, Cell growth, LNK, 14-3-3 ε/γ

## Abstract

**Background:**

Rapid progression contributes to treatment failure in anaplastic thyroid carcinoma (ATC) patients. In a preliminary study, we demonstrated that some hematopoietic factors may be involved in the progression of ATC. The adaptor protein LNK, which is a negative regulator of hematopoietic cytokine signalling, has been studied extensively in malignant hematopoietic cells. However, there are few studies on LNK in solid tumours.

**Methods:**

Real-time PCR, immunohistochemistry (IHC) and western blot analysis of LNK were performed on ATC cells, differentiated thyroid cancer (DTC) cells and normal thyroid cells. In vitro assays (including pull-down, liquid chromatography-mass spectrometry (LC–MS), co-IP, MTT and colony formation) were performed to validate the effect of LNK on ATC progression and elucidate the molecular mechanisms.

**Results:**

Compared with DTC cells and normal thyroid cells, ATC cells exhibit overexpression of LNK. In addition, LNK overexpression results in increased proliferation of ATC cells. Conversely, LNK knockdown significantly suppresses ATC cell proliferation. LC–MS identified the 14-3-3 ε/γ protein as a LNK binding partner. Finally, the results indicate that LNK overexpression significantly enhances the anti-apoptotic ability of ATC cells via the Akt-NFκB-Bcl-2/Bcl-xL pathway and that the oncogenic effect of LNK largely depends on 14-3-3 ε/γ binding.

**Conclusions:**

The present study elucidated the important role of LNK in the growth of ATC opposite to its behaviour in the hematopoietic system and indicates that LNK is a potential target for the treatment of ATC.

## Background

Anaplastic thyroid carcinoma (ATC) is one of the most aggressive cancers in humans. Although it accounts for 1–2% of all thyroid cancers, it is responsible for the majority of thyroid carcinoma-related deaths [1, 2]. A lack of early symptoms and a late diagnosis contribute to its poor prognosis, with most patients presenting with terminal cancer upon diagnosis. Due to a lack of effective treatment measures, the 1-year overall survival rate of ATC patients after diagnosis is only 20%, and the median survival time is 3–9 months [1–3]. Therefore, exploring the mechanism underlying the rapid progression of ATC and finding new molecular therapeutic targets are very important for improving the prognosis of ATC patients.

Previously, we found that increased white blood cell and platelet counts are negatively correlated with the prognosis of ATC patients [4]. Furthermore, we found that the hematopoietic factors interleukin-11 and colony-stimulating factor-1 significantly increased the invasive and migratory abilities of ATC cells [4, 5]. Therefore, we speculated that some genes that regulate hematopoietic factors are involved in the progression of ATC. Recent research has shown that functional deletion mutations in LNK, an important hematopoietic suppressor gene, lead to a > 10-fold increase in hematopoietic stem cell numbers owing to superior hematopoietic stem cell self-renewal [6] and gives rise to myeloproliferative neoplasms characterized by platelet and leukocyte overproduction [7].

LNK, also known as SH2B3, is a member of the SH2B family of adaptor proteins primarily expressed in hematopoietic cells. It contains a pleckstrin homology (PH) domain and a Src homology 2 (SH2) domain that specifically bind to phosphorylated tyrosine residues, which mediates signal transduction, and an N-terminal proline-rich region that mediates dimerization [8, 9]. Studies have shown that LNK can inhibit wild-type or mutant JAK2 signal transduction through the SH2 domain and inhibit the activation of the JAK/STAT, ERK/MAPK, and PI3K/Akt/mTOR/GSK3β pathways [9–13]. Clinical studies have found that LNK mutations can lead to diabetes, heart disease, kidney injury, autoimmune hepatitis, acute lymphocytic leukemia, and bone marrow proliferative malignancies [6, 13–18].

Most of the studies that have explored the role of LNK have focused on haematologic cells. In these cells, LNK downregulation activated tyrosine kinases at the cell surface, resulting in an anti-proliferative effect in the hematopoietic system [11, 19]. However, there are few studies on LNK in solid tumors. It was found that LNK expression was upregulated in high-grade ovarian cancer and acted as a positive signal transduction modulator, opposite to its behavior in the hematopoietic system [8]. Therefore, we hypothesized that LNK, a hematopoietic-factor suppressor gene, is associated with the rapid progression of ATC. Herein, on the basis of previous studies, we explore the biological function and mechanism of LNK in ATC, which will provide new ideas and targets for the treatment of ATC.

## Materials and methods

### Clinical tissue specimens

The tissue samples were collected and were used in accordance with approval by the Ethics Committee of the Third Affiliated Hospital of Kunming Medical University. Eleven cases of ATC, DTC and normal thyroid tissues were selected for detecting mRNA level. All these tissues were collected from patients who had undergone surgical resection at the Third Affiliated Hospital of Kunming Medical University between May 2015 and May 2018. The fresh tissues were immediately stored at liquid nitrogen container until RNA extraction was performed.

### Cell lines and cell culture

Human thyroid follicular epithelial cell line (Nthy-ori-3-l) and Human papillary thyroid cancer (PTC) cell lines (KAT-5, B-CPAP, KTC-1), ATC cell lines (ARO, FRO, sw579) and human embryonic kidney 293FT cell lines were purchased from the American Type Culture Collection (ATCC, Manassas, VA, USA). The ATC cell line (8305C) was purchased from the European Collection of Cell Cultures (ECACC, Salisbury, United Kingdom). All cells were cultured in DMEM supplemented with 10% fetal bovine serum (FBS, USA), penicillin (100 U/ml) and streptomycin (100 U/ml) at 37 °C in a humidified 5% CO_2_ incubator.

### Antibodies and reagents

The following antibodies were used in this study: primary antibodies against LNK and α-tubulin were purchased from Sigma-Aldrich (St. Louis, MO, USA); primary antibodies against GAPDH, p-Akt (Ser473), Akt, NF-κb(P50), Bcl-2, Bcl-xl, Caspase 7, Cleaved Caspase 7, Caspase 9, Cleaved Caspase 9 and 14-3-3 were purchased from Cell signal technology (Danvers, MA,USA). Unless otherwise indicated, all other reagents were purchased from Sigma-Aldrich (St. Louis, MO, USA).

### Western blotting

Cells were washed with PBS and then lysed in sodiumdodecyl sulfate (SDS) sample buffer. The protein concentration in the lysate was measured by a BCA protein assay. A total of 30 μg protein was separated on a 9% SDS–polyacrylamide gel by electrophoresis, and transferred to a polyvinylidene fluoride (PVDF) membrane for 3 h at 250 mA, and blocked with 5% skim milk for an hour, then incubated with primary antibodies at 4 °C overnight. Followed by incubation with secondary antibodies at room temperature for 50 min. After three times wash, bound antibodies were visualized via electrochemiluminescence (ECL), which was captured by XAR film. Scanning and analysis of the western blot bands were performed by the Quantity One program (Bio-Rad, USA).

### Immunohistochemistry (IHC)

Paraffin-embedded ATC, DTC and normal thyroid tissue specimens, which were pathologically and clinically diagnosed at the Third Affiliated Hospital of Kunming Medical University, were sliced 4-μm-thick sections and incubated at 60 °C for 2 h. Immunohistochemical assays were performed as previously described [5]. All slides were independently assessed by two pathologists who were blinded to the patients’ identities and clinical outcomes. Tissues were considered positive expression when cytoplasmic staining was detected, and the intensity of the cells expressing LNK or 14-3-3 was scored as follows: 0 (no or weak staining, light yellow), 1 (moderate staining, yellow–brown) or 2 (strong staining, brown). The percentage of positive staining was scored as follows: 0 (no expression), 1 (1–25%), 2 (26–75%), 3 (> 75%). The LNK or 14-3-3 expression level was calculated as the intensity score plus the proportion score and was divided into 4 grades: − (score of 0 and 1), + (score of 2), ++ (score of 3) and +++ (score of 4 and 5). In the present study, ‘−’ and ‘+’ represented no/low expression (score of 0–2), whereas ‘++’ and ‘+++’ were regarded as high expression (score of 3–5).

### Stable cell line generation

For the LNK overexpression plasmid, cDNA comprising the full-length coding region of LNK cDNA was amplified by PCR, and the digested and purified PCR products were directly cloned into a pMSCV.puro.retroviral vector. For LNK knockdown, lentivirus particles were produced in 293FT cells by transfection of pLKO.1-puro plasmid harboring specific shRNAs, the sequences of which were created with Invitrogen’s siRNA design tool (Invitrogen, Carlsbad, CA, USA). A standard calcium phosphate cotransfection procedure was performed with a PIK packaging plasmid in the 293FT cells as previously described [5]. The target cells were incubated with their respective virus in 2 µg/ml Polybrene (Sigma-Aldrich) to promote infection. Twenty-four hours after infection, the cells were incubated with 1 µg/ml puromycin for 3 days to select cells with stable integration of the vectors.

### Quantitative real-time polymerase chain reaction (real-time PCR)

Total RNA was isolated from ATC cells using TRIzol Reagent, and 2 μg of each RNA sample was reverse-transcribed using M-MLV Reverse Transcriptase (Invitrogen, USA). Fast SYBR GreenMaster Mix was used to analyse the threshold cycle value of each sample by a qRT-PCR detection system (Bio-Rad, USA). GAPDH was used as an internal control to normalize the expression levels. The following primer pairs were used: LNK, sense, 5′-TGTGAGTTGCACGCCGTAGC-3′, and antisense, 5′-GCACCTCGCGGCAGAAGTAG-3′; 14-3-3ε, sense, 5′-GATTCGGGAATATCGGCAAATGG-3′, and antisense, 5′-GCTGGAATGAGGTGTTTGTCC-3′; and 14-3-3γ, sense, 5′-AGCCACTGTCGAATGAGGAAC-3′, and antisense, 5′-CTGCTCAATGCTACTGATGACC-3′. PCR was performed on a PTC-200 PCR system (Bio-Rad, USA) using the following cyclical procedure: 10 min at 95 °C; followed by 40 cycles of 10 s at 95 °C, 10 s at 55 °C, 20 s at 72 °C; and a final extension at 72 °C for 10 min. The reactions were run in triplicate in three independent experiments.

### MTT assay

Cells were seeded in 96-well culture plates at a density of 1 × 10^3^ cells/well in triplicate. The culture plates were then harvested at the indicated times. Then, 20 μl of 5 mg/ml MTT was added to each well, followed by incubation for 4 h. The culture medium was discarded, and 150 μl of dimethyl sulfoxide (DMSO) was added to each well. The optical density (OD) at 490 nm was measured with a micro-culture plate reader (Bio-Rad, USA). The absorbance values were normalized to the control wells, and proliferation was expressed as the percentage of the control. Each experiment was performed in three independent experiments.

### Colony formation assay

ATC cells were seeded in 6-well culture plates at a density of 200 cells/well in triplicate. The culture medium was changed every 2 days. After 10 days, the resulting colonies were fixed in methanol and stained with 0.5% crystal violet, after which they were photographed and counted. Colonies containing more than 100 cells were counted.

### Co-immunoprecipitation

LNK-overexpressing ATC cell lines were lysed in a protein lysis buffer (20 mM Tris–HCl (pH 7.5), 150 mM NaCl, 1 mM Na_2_EDTA, 1 mM EGTA, 1% Nonidet P-40, 1% sodium deoxycholate, 2.5 mM sodium pyrophosphate, 1 mM β-glycerophosphate, 1 mM Na_3_VO_4_, and 1 μg/ml leupeptin with protease inhibitor cocktail and phosphatase inhibitors) at 4 °C for 30 min and centrifuged at 13,000 rpm (10 min, 4 °C) to remove cell debris. For immunoprecipitation, antibodies against FLAG or myc were added to the lysates and incubated overnight at 4 °C, with rabbit IgG (1:100) serving as a control antibody. Then, protein A/G agarose beads were added and incubated with the lysates for 1 h at 4 °C. After the beads were washed with protein lysis buffer 5 times, any remaining bound proteins were eluted in protein loading buffer and analyzed by immunoblotting.

### FLAG pull-down assay and silver staining

293FT cells were transfected with a pMSCV empty vector or the pMSCV-LNK-flag plasmid. Thirty-six hours later, the cells were treated with IP lysis buffer (150 mM NaCl; 1% NP-40) on ice for 30 min. The protein was collected and centrifuged to discard any precipitates. Then, 30 µl of FLAG beads (Sigma) was added to each of samples and mixed at 4 °C overnight. The next day, the supernatant was centrifuged and discarded, and the beads were washed 5 times with IP buffer. Then, 30 μl of FLAG peptide (200 µg/ml, Sigma) was added, and the mixture was shaken for 2 h. Next, the supernatant was centrifuged and denatured, and the SDS-PAGE was performed. After electrophoresis, the gel was released, and fixed for an hour, and then washed with 30% ethanol for 10 min followed by a sensitizer for 2 min. After the gel was washed with ultrapure water 2 times, a silver solution as added for 10 min. Destaining was performed, and the reaction was terminated when the expected bands appeared. The protein bands present were subsequently used for liquid chromatography–mass spectrometry (LC–MS) analysis.

### Annexin V/PI apoptosis assay

After the ATC cell line ARO was harvested and washed twice with PBS, the cells were resuspended in 400 μl of binding buffer containing 10 μl of APC-labeled Annexin-V and 5 μl of PI solution (4A Biotech co., China). Cells were kept on ice for 10 min and subjected to flow cytometry (Beckman) to assess the percentage of apoptotic cells.

### Statistical analysis

All statistical analyses were conducted using SPSS statistical package 19.0 (SPSS Inc., Chicago, IL, USA). Differences between two groups were analyzed by Student’s t test. ANOVA was performed to compare the differences among 3 or more groups. *P < 0.05, **P < 0.01, and ***P < 0.001 were indicative of statistically significant results as shown in the figures. Error bars in the experiments indicate the standard error of the mean (SEM) or standard deviation (SD) for a minimum of three independent experiments.

## Results

### ATC cells exhibit increased LNK expression

**Fig. 1:**
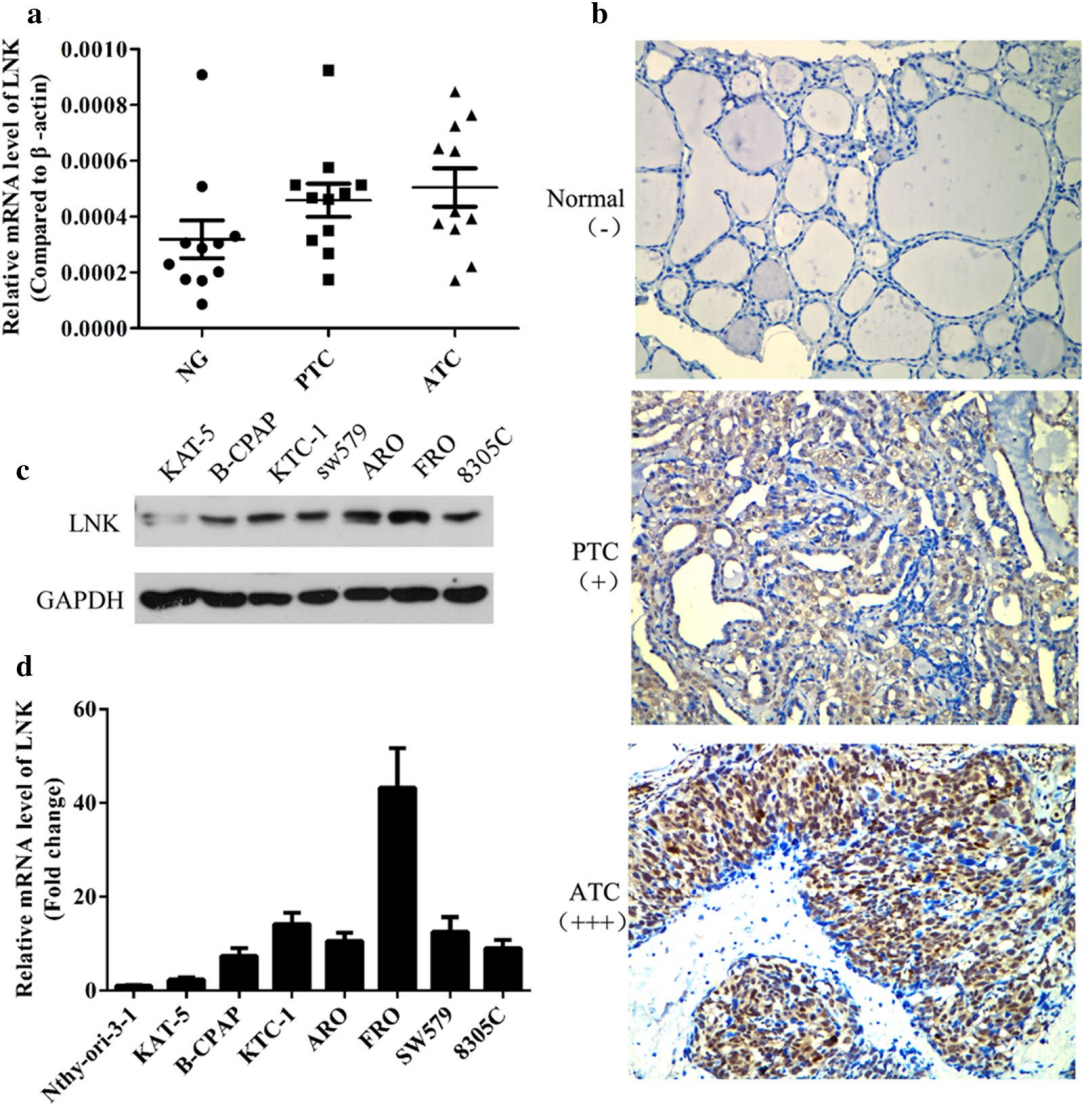
ATC cells exhibit increased LNK expression. **a** The mRNA and **b** protein expression levels of LNK were significantly higher in ATC tissues than in DTC and normal thyroid tissues, as measured by real-time PCR and IHC, respectively. **c** The protein and **d** mRNA expression levels of LNK were significantly higher in the ATC cell lines (ARO, FRO, SW579, 8305C) than in the DTC cell lines (KTC-1, KAT-5, B-CPAP) and a thyroid follicular epithelial cell line (Nthy-ori-3-1), as measured by western blot and real-time PCR, respectively

To explore LNK expression in thyroid carcinoma cells at different pathological grades, real-time PCR and IHC were performed to assess LNK expression in ATC, DTC and normal thyroid tissues. The real-time PCR results showed that the LNK mRNA expression was higher in ATC tissues than in DTC and normal thyroid tissues (Fig. 1a). Consistent with this, the IHC results showed a similar tendency (Fig. 1b). Moreover, in examining LNK expression in the cell lines, western blot results revealed higher LNK protein expression in the ATC cell lines (ARO, FRO, SW579, 8305C) than in the DTC cell lines (KTC-1, KAT-5, B-CPAP) and a thyroid follicular epithelial cell line (Nthy-ori-3-1) (Fig. 1c). Similar results were also found for LNK mRNA expression based on real-time PCR (Fig. 1d). Thus, our data indicated that the mRNA and protein expression levels of LNK were significantly increased in the ATC cells compared with those in the DTC and normal thyroid cells.

### LNK is closely associated with the growth of ATC cells

**Fig. 2:**
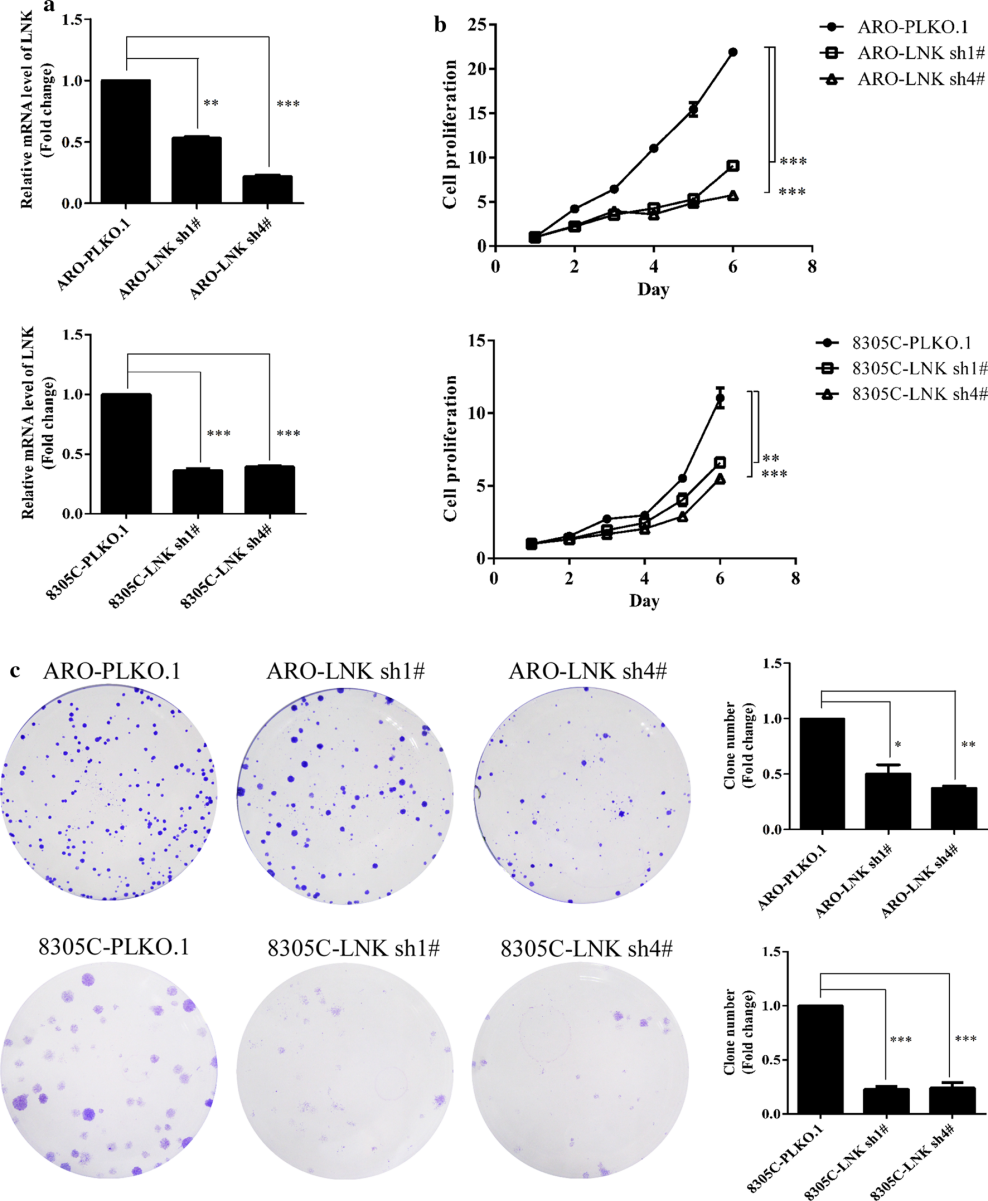
Effects of LNK knockdown on ATC cell growth. **a** After LNK was knocked down, the mRNA levels of LNK in ARO and 8305C cells significantly decreased, as measured by real-time PCR. After knockdown of LNK, the proliferation abilities of ATC cells were significantly decreased compared to those of the NC cells, as assessed by the **b** MTT and **c** colony formation assays. *P < 0.05, **P < 0.01, ***P < 0.001

To clarify the role of LNK in ATC cell growth, we generated ATC cells with stable knockdown of LNK expression using retroviral vectors (LNK-sh1#, 2#, 3# and 4#), with scrambled shRNA serving as the negative control (NC). Real-time PCR and western blot were performed to assess LNK knockdown efficiency. As shown in Additional file 1: Figure S1 and Fig. 2a, LNK mRNA and protein expression levels were significantly lower in LNK-sh cells than in NC cells, especially in the sh1# and sh4# cells; therefore, we chose LNK-sh1# and LNK-sh4# ATC cells for all subsequent experiments. Then, we performed MTT assays and colony formation assays to detect ATC cell proliferation. We found that after LNK knockdown, the proliferative capacities of ARO and 8305C cells were decreased (Fig. 2b), and the number of colonies formed was significantly reduced (Fig. 2c).

**Fig. 3:**
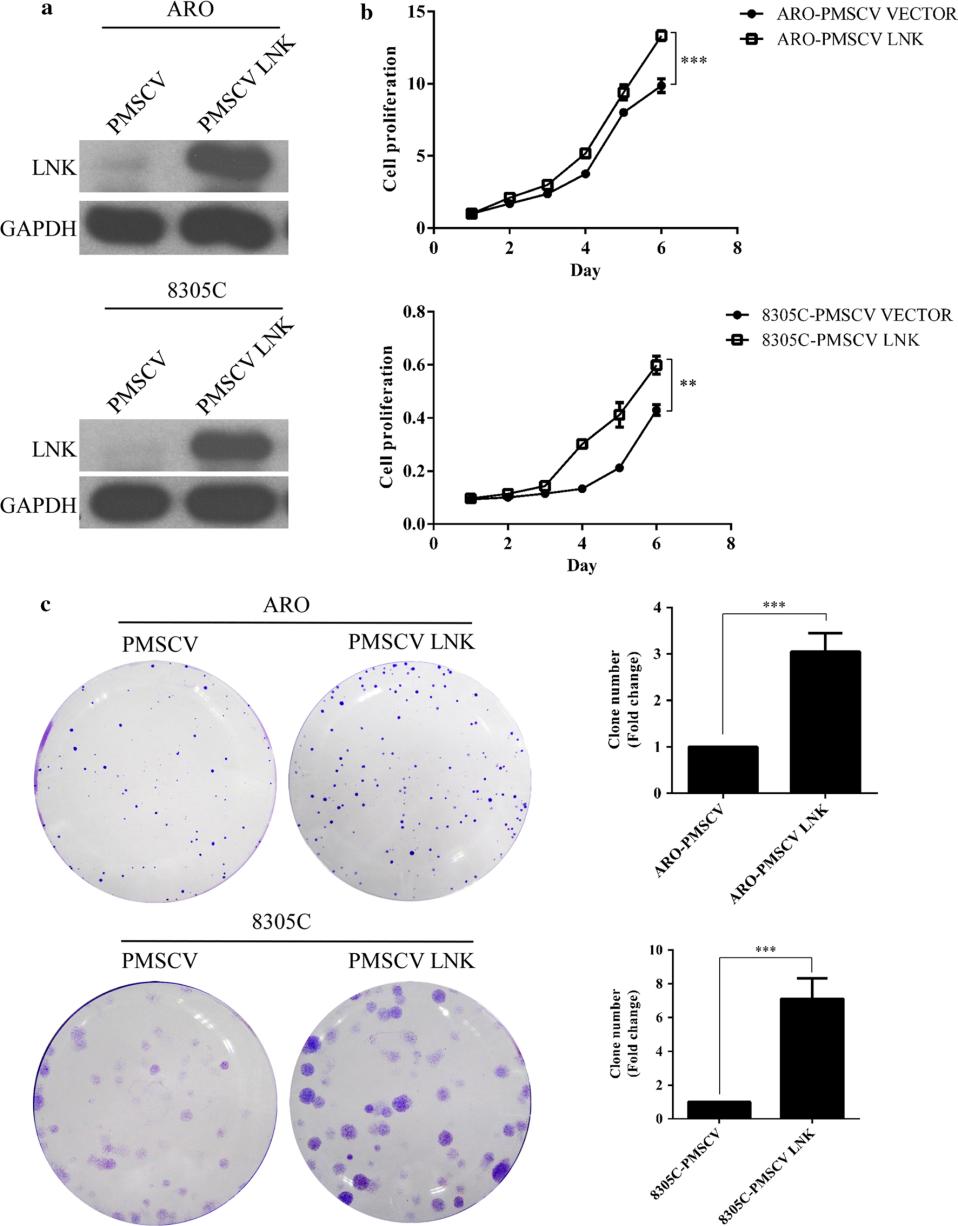
Effects of LNK overexpression on ATC cell growth. **a** Upon LNK overexpression, the protein levels of LNK in ARO and 8305C cells significantly increased, as measured by western blot. In addition, the proliferation abilities of ATC cells with LNK overexpression were significantly increased compared to those of the NC cells, as assessed by the **b** MTT and **c** colony formation assays. *P < 0.05, **P < 0.01, ***P < 0.001

To further confirm the role of LNK in ATC cell growth, we constructed ATC cells with stable overexpression of LNK using a retroviral vector. The western blot results showed that the LNK protein expression was higher in pMSCV-LNK ATC cell lines than that in NC cells (Fig. 3a). MTT assays and colony formation assays were performed to measure cell proliferation. As shown in Fig. 3b, c, ATC cells with LNK overexpression show greater proliferation capabilities than NC cells. These results further confirmed the growth-promoting effect of LNK in ATC cells.

### LNK directly interacts with 14-3-3ε/γ

**Fig. 4:**
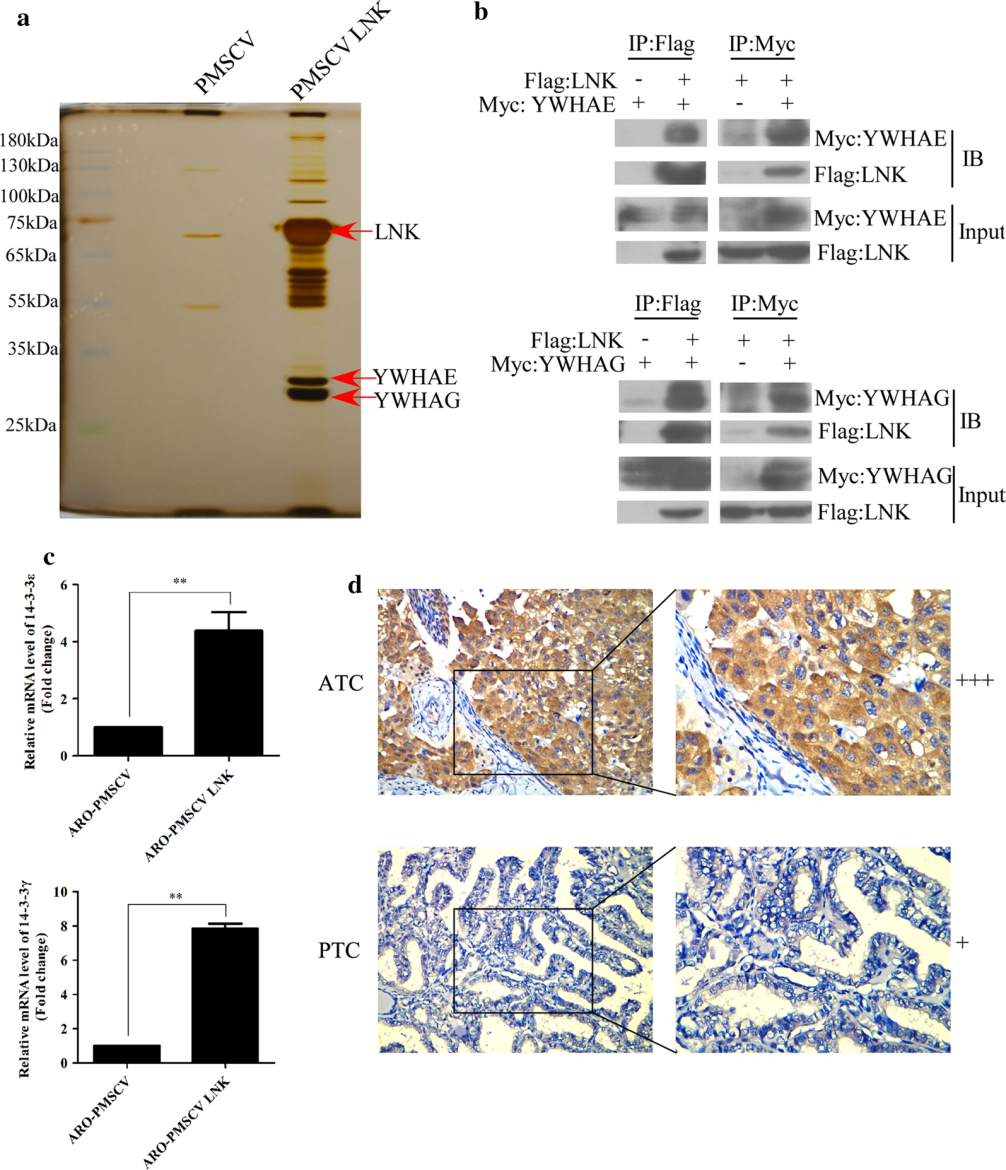
LNK interacts with 14-3-3ε/γ (YWHAE/YWHAG) in ATC cells. **a** Potential LNK binding partners identified by a FLAG pull-down assay combined with LC–MS. **b** Co-IP assays confirmed LNK binding to 14-3-3ε/γ (YWHAE/YWHAG) in ATC cells. 14-3-3 proteins were tagged with myc, and LNK was tagged with FLAG. **c** Real-time PCR showed that the mRNA expression levels of 14-3-3ε and 14-3-3γ were increased in LNK-overexpressing ARO cells. **d** The protein expression level of 14-3-3 in paraffin-embedded ATC and PTC tissue specimens as measured by IHC. +++: high expression, +: low expression. Original magnification, ×40

To further investigate the potential mechanism by which LNK regulates signal transduction in ATC, we used a FLAG pull-down assay combined with silver staining to explore the potential interacting proteins of LNK in 293FT cells. Intriguingly, we found two unique bands, which we processed and examined with LC–MS; the results showed that 14-3-3ε (YWHAE) and 14-3-3γ (YWHAG) may bind with LNK (Fig. 4a). Next, a co-IP (co-immunoprecipitation) assay was performed to confirm whether 14-3-3ε and 14-3-3γ were binding partners of LNK in ATC cells (Fig. 4b).

The mRNA expression levels of 14-3-3ε and 14-3-3γ were increased in LNK-overexpressing ARO cells (Fig. 4c). In addition, IHC was performed and showed that the protein expression levels of 14-3-3ε and 14-3-3γ in ATC tissues were higher than those in DTC tissues (Fig. 4d).

### Knockdown of 14-3-3ε/γ reverses the growth-promoting effect of LNK in ATC

**Fig. 5:**
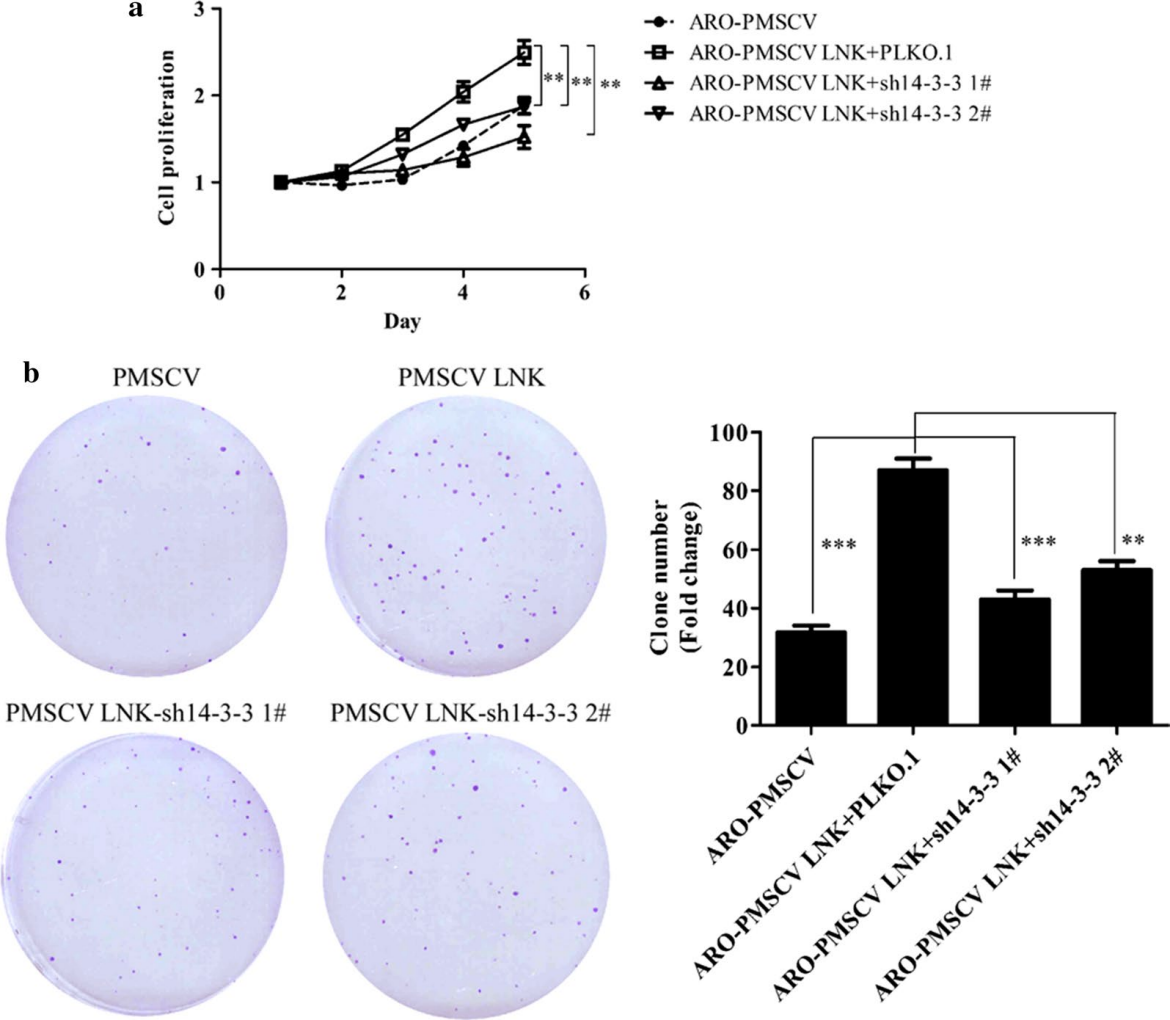
LNK-mediated promotion of ATC cell proliferation is depending on the 14-3-3 proteins. MTT (**a**) and colony formation (**b**) assays showed that the enhanced proliferation and colony-forming abilities of ATC cells induced by LNK overexpression were offset by knocking down 14-3-3ε/γ

After confirming the interaction between LNK and 14-3-3ε/γ, we further explored the role of the interaction between the 14-3-3 proteins and LNK in ATC growth. We constructed the recombinant plasmids pLKO.1-sh14-3-3ε and pLKO.1-sh14-3-3γ, which were enveloped in lentiviruses and transduced into ARO cells with stable LNK overexpression. After puromycin treatment for 3 days, a LNK-overexpressing ARO cell line with 14-3-3ε/γ knockdown was obtained. As shown in Fig. 5a, b, the enhanced proliferation and cloning abilities of ATC cells induced by LNK overexpression were offset by the knockdown of 14-3-3ε/γ. These results confirmed that the growth-promoting effect of LNK in ATC partially depends on 14-3-3ε/γ.

### LNK -mediated augmentation of the anti-apoptotic ability of ATC cells may depend on the Akt-NFκB-Bcl-2/Bcl-xL pathway

**Fig. 6:**
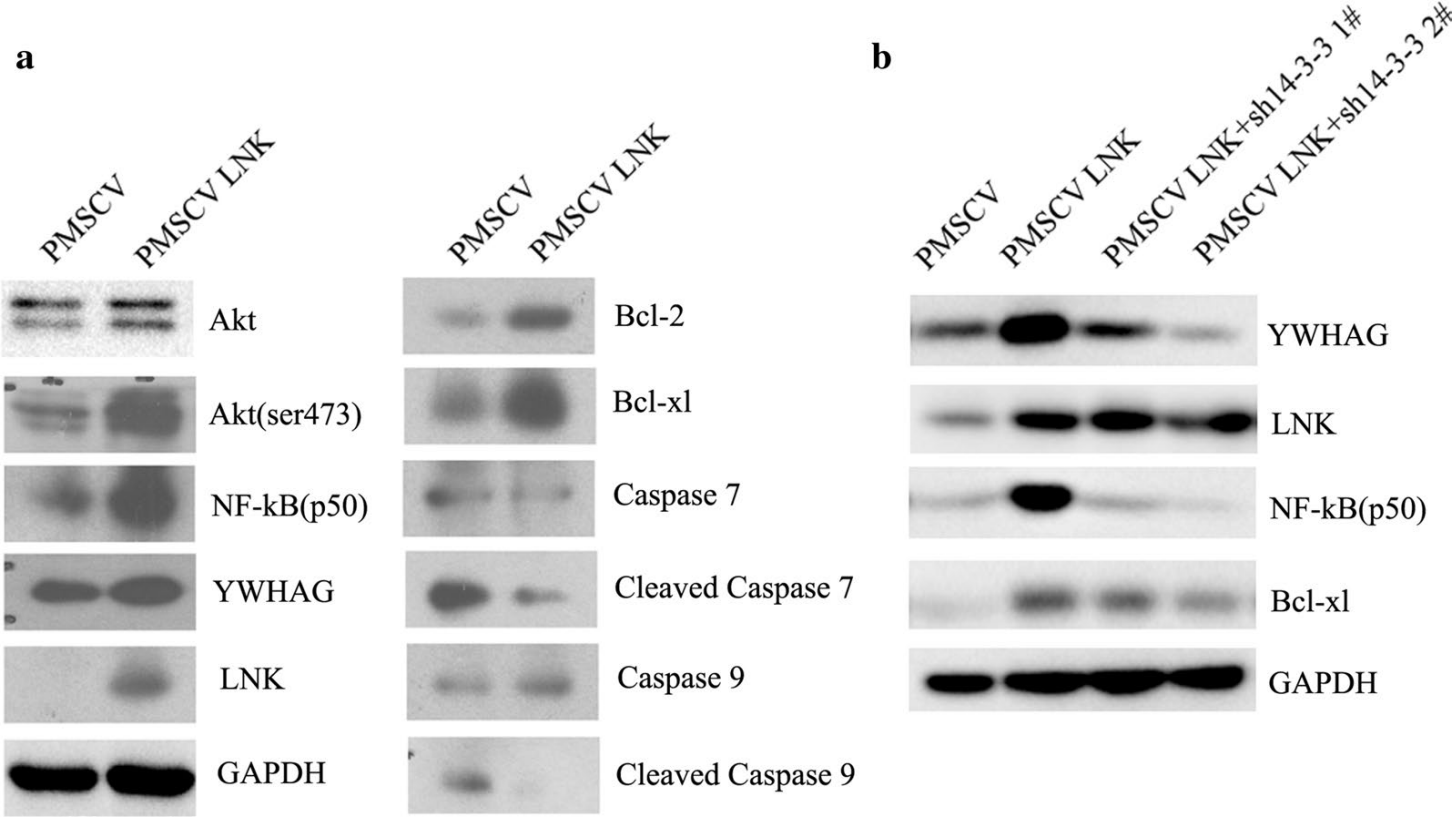
LNK promotes the anti-apoptotic ability of ATC cells by activating the Akt-NFκB-Bcl-2/Bcl-xL pathway. **a** Protein expression levels of constituents of a number of signaling pathways in ARO cells with ectopic LNK expression. Total proteins were separated by SDS-PAGE and subjected to western blotting with the indicated antibodies. **b** Western blot analysis of the protein expression of members of several signaling pathways in LNK-overexpressing ARO cells with stable knockdown of 14-3-3 protein via shRNA

Previous studies have demonstrated that 14-3-3 could promote cell proliferation in several cancers by inhibiting apoptosis via activation of the Akt pathway [20, 21]. Since Akt activation has emerged as an important feature of anti-apoptosis activity, we asked whether the interaction between LNK and 14-3-3ε/γ also inhibited apoptosis in ATC cells by regulating Akt activity. As shown in Fig. 6a, we found that LNK overexpression caused elevated expression of the 14-3-3 proteins accompanied by elevated levels of phosphorylated Akt (Ser475) in ATC cells. Meanwhile, the protein expression levels of anti-apoptotic proteins (Bcl-2, Bcl-xL and NFκB) were increased, and the activation of the pro-apoptotic protein protease 7/9 (caspase-7/9) was downregulated (Fig. 6a). In addition, we further found that the anti-apoptotic effect of LNK overexpression could be partially blocked with 14-3-3 knockdown (Fig. 6b). Furthermore, the Annexin V/PI apoptosis assay results showed that LNK knockdown increased apoptosis in ATC cell lines (Additional file 1: Figure S2). Taken together, these findings provide strong support that LNK can activate the Akt-NFκB-Bcl-2/Bcl-xL anti-apoptotic pathway, thus promoting cell survival and growth in ATC, and that this biological process largely depends on 14-3-3ε/γ.

## Discussion

Despite improvements in surgical methods, radiotherapy, chemotherapy and other cancer treatment strategies, the median survival time of ATC patients is 3–9 months [1–3]. There is a lack of effective therapies to control the high proliferation and mortality of ATC. Therefore, there is an urgent need to develop new molecular therapeutic targets to improve the prognosis of ATC patients.

LNK is an adaptor protein that has been extensively studied in normal and malignant hematopoietic cells. In these cells, it downregulates activated tyrosine kinases at the cell surface, resulting in an anti-proliferative effect [8]. Previously, it was confirmed that LNK mutations lead to functional deficiency, which is closely related to the occurrence and development of hematological malignancies, especially in Philadelphia chromosome-like acute lymphoblastic leukemia (Ph-like ALL) and myeloproliferative neoplasms [11, 22–24]. Research has also shown that LNK inhibits the hematopoietic stem cell/progenitor cell response to various cytokines by inhibiting the JAK2 signaling pathway [13, 22]. Furthermore, LNK can also inhibit the progression of radiation-induced acute B-cell malignant tumors in mice. Moreover, hematopoietic stem cells with LNK deficiency can recover from radiation more effectively than can their wild-type counterparts [9, 25]. The hematopoietic cells of LNK-knockout mice were highly sensitive to a series of cytokines, including IL-3, IL-7, EPO, and TPO [25]. As a result, the JAK/STAT and ERK/MAPK pathways are highly activated [9, 10]. Mice with LNK deficiency exhibited hematopoietic disorders, including a triple increase in circulating white blood cell and platelet levels, the accumulation of immature B cells in bone marrow and spleen, and the expansion of hematopoietic stem cell pools with an enhanced self-renewal ability via the JAK2 pathway [19].

However, the study of LNK in solid tumors is relatively rare. Ding et al. [8] found that the prognosis of ovarian cancer patients with high LNK expression is poor, and that LNK expression is higher in ovarian cancer with poorer differentiation. LNK overexpression rendered ovarian cancer cells resistant to death and generated larger tumors in a murine xenograft model. Their results suggested that LNK plays a role as an oncogene in ovarian cancers, in contrast to the findings in hematologic disorders [8].

LNK plays an important role in the regulation of the hematopoietic stem cell/progenitor cell response to various cytokines. On the other hand, white blood cell and platelet counts as well as several hematopoietic factors are closely related to the prognosis of patients with ATC and to the invasion and metastatic abilities of ATC cells [4, 5, 26]. Therefore, it is highly valuable to explore the function and mechanism of LNK in the progression of ATC. We first explored the LNK expression in thyroid tumor tissues, and found that the higher the degree of malignancy of thyroid tumors was, the higher the expression level of LNK. Therefore, we preliminarily speculated that LNK may be related to the rapid growth of ATC. To verify this hypothesis, we stably knocked down LNK in ATC cell lines (ARO, 8305C). The results showed that after stably interfering with LNK expression, the proliferation ability of these ATC cell lines was significantly weakened. In contrast, in cells with stable overexpression of LNK, the above malignant biological behaviors were significantly enhanced. These results suggest that LNK may play a similar oncogenic role in ATC as it does in ovarian cancer.

To further investigate the potential mechanisms underlying the role of LNK in ATC, we used a FLAG pull-down assay and LC–MS to analyze the binding proteins of LNK in ATC cells. As a result, we found that LNK could bind to the 14-3-3ε and 14-3-3γ proteins, which was also confirmed by co-IP experiments. Furthermore, we knocked down the expression of 14-3-3ε/γ in cells with stable LNK overexpression and found that the proliferation ability of LNK-overexpressing ATC cells was significantly weakened after knocking down 14-3-3ε/γ. Previous studies have also found that 14-3-3 protein binding with LNK weakens the inhibitory effect of LNK on the JAK2 pathway and on cell proliferation in hematopoietic stem cells [21, 27]. Ding et al. [8] also found that 14-3-3 protein binding with LNK inhibited the carcinogenic effect of LNK in ovarian cancer cells. These results suggest that LNK binding with 14-3-3 protein is important in the functioning of LNK in solid tumors.

At present, many studies have indicated that the 14-3-3 protein plays an oncogenic role in many kinds of tumors and inhibits apoptosis [20, 28–30]. Therefore, we focused on monitoring the expression level of apoptosis-related proteins after stable overexpression of LNK. The results showed that LNK overexpression could cause an elevated expression of 14-3-3 proteins accompanied by increased levels of phosphorylated Akt, and enhance the expression of the anti-apoptotic proteins Bcl-2, Bcl-xL and NFκB and downregulate the activation of the pro-apoptotic protein caspase-7/9. Moreover, this series of expression changes was partially blocked by knocking down 14-3-3 protein expression. These results indicated that LNK can activate the Akt-NFκB-Bcl-2/Bcl-xL anti-apoptotic pathway in ATC, thus promoting cell survival and growth, and this biological process depends on the presence of 14-3-3 ε/γ.

## Conclusions

Altogether, our results showed, for the first time, that LNK overexpression results in increased proliferation and anti-apoptotic signaling by activating the Akt-NFκB-Bcl-2/Bcl-xL pathway, and its oncogenic effect largely depends on 14-3-3 ε/γ proteins. The present study elucidated the important role played by LNK in ATC growth and indicates that LNK is a potential target for the treatment of ATC.

## Supplementary information

**Additional file 1: Figure S1.** Successful construction of recombinant LNK-shRNA plasmids. (A) LNK mRNA expression in LNK-shRNA cells (sh1#, sh2#, sh3# and sh4#) and control cells (PLKO.1) were measured by Real-time PCR analysis. (B) Western blot analysis was used to examine the protein expression of LNK-shRNA cells and control cells.** Figure S2.** LNK knockdown increased cell apoptosis in ARO cells. Annexin V-PI apoptosis assays were used to examine the cell apoptosis level in ARO-PLKO.1 (A) and LNK-shRNA cells (B and C).

## Data Availability

All data generated or analyzed during this study are included in this published article.

## Abbreviations

ATC: anaplastic thyroid carcinoma
DTC: differentiated thyroid carcinoma
PTC: papillary thyroid carcinoma, which is the main type of DTC
LC–MS: liquid chromatograph–mass spectrometer
IHC: immunohistochemistry
DMEM: Dulbecco’s modified eagle’s medium
FBS: foetal bovine serum
SDS: sodiumdodecyl sulfate
ECL: electrochemiluminescence
PVDF: polyvinylidene fluoride film
IP: immunoprecipitation
Bcl-2: B cell lymphoma-2
NFκB: nuclear factor-kappa B
