# Supplementary material for: Adaptor protein LNK promotes anaplastic thyroid carcinoma cell growth via 14-3-3 ε/γ binding
Source: Cancer Cell Int. 2020 Jan 9;20:11. doi: 10.1186/s12935-019-1090-9 (PMC6953139; doi:10.1186/s12935-019-1090-9)

Additional Figure legends

Additional Figure S1. Successful construction of recombinant LNK-shRNA plasmids. (A) LNK mRNA expression in LNK-shRNA cells (sh1#, sh2#, sh3# and sh4#) and control cells (PLKO.1) were measured by Real-time PCR analysis. (B) Western blot analysis was used to examine the protein expression of LNK-shRNA cells and control cells.


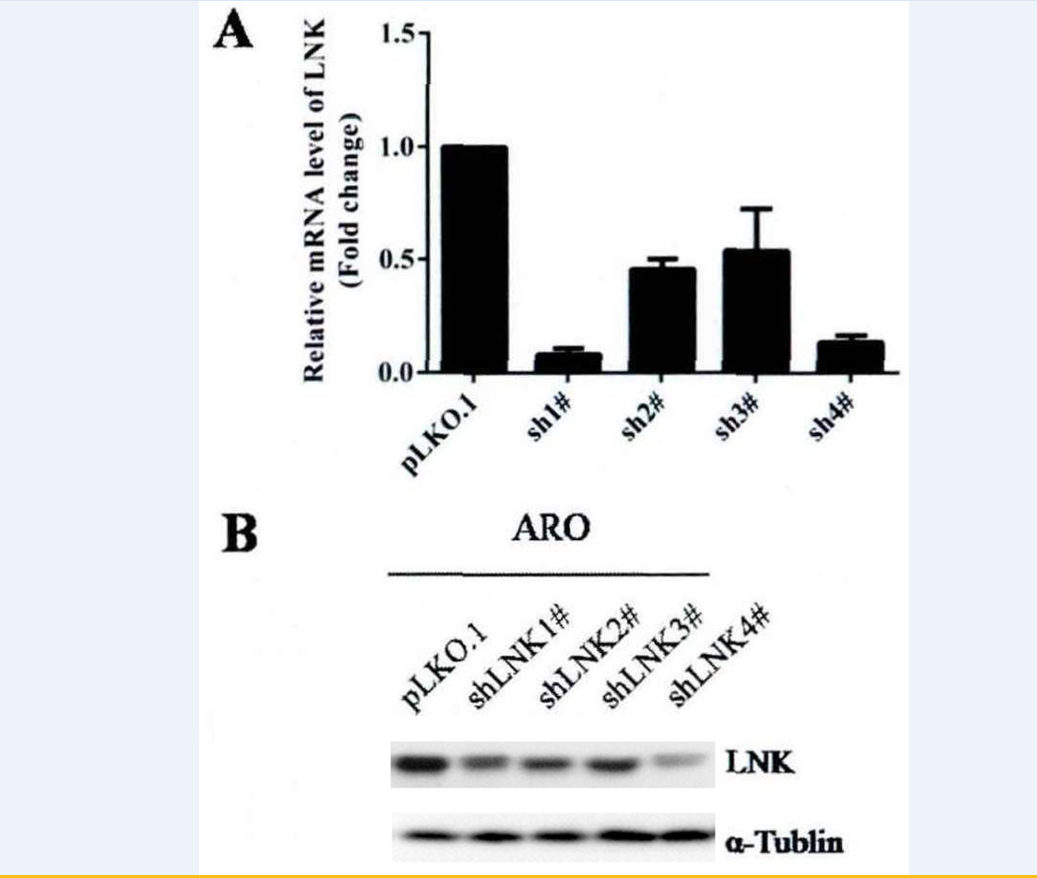


Additional Figure S2. LNK knockdown increased cell apoptosis in ARO cells. Annexin V-PI apoptosis assays were used to examine the cell apoptosis level in ARO-PLKO.1 (A) and LNK-shRNA cells (B and C).


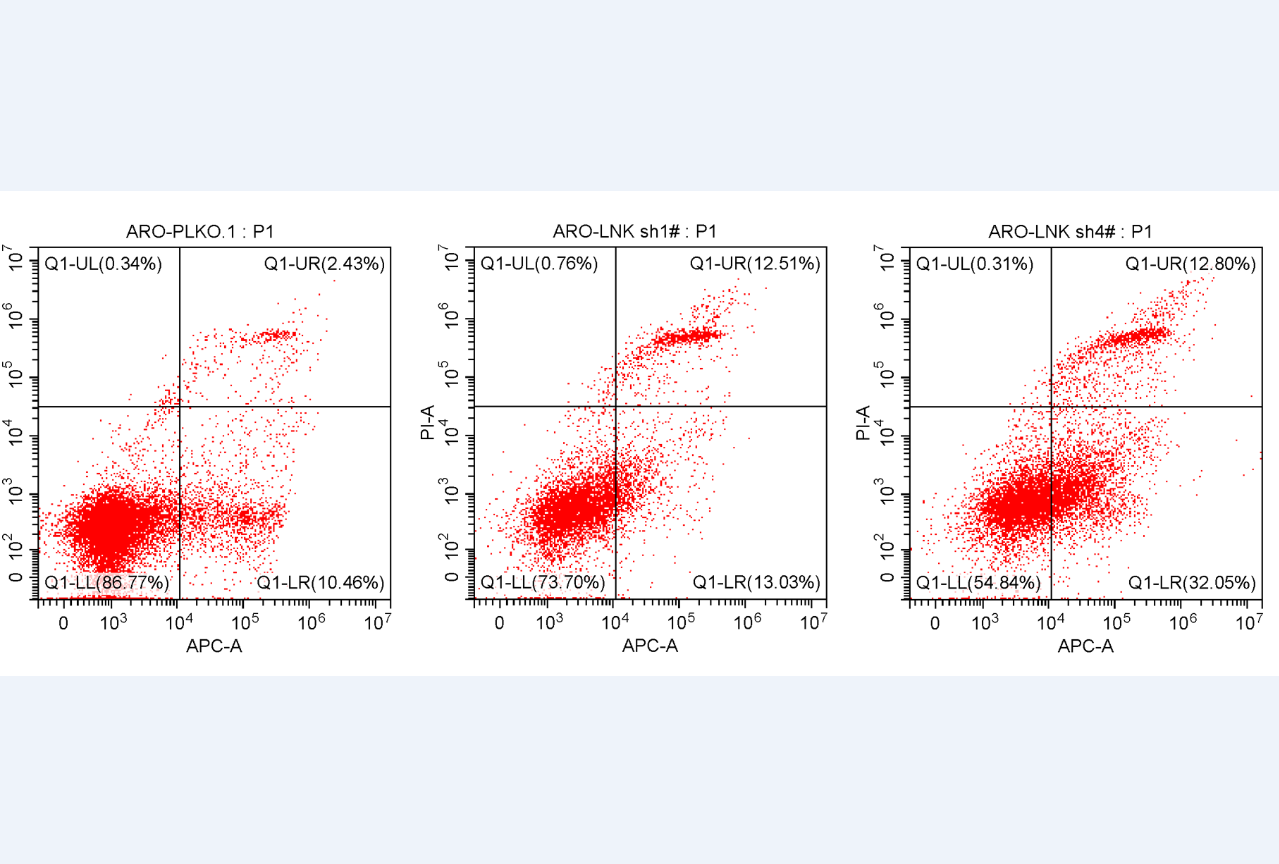

Supplement: Supplementary file 1 — Additional file 1: Figure S1. Successful construction of recombinant LNK-shRNA plasmids. (A) LNK mRNA expression in LNK-shRNA cells (sh1#, sh2#, sh3# and sh4#) and control cells (PLKO.1) were measured by Real-time PCR analysis. (B) Western blot analysis was used to examine the protein expression of LNK-shRNA cells and control cells. Figure S2. LNK knockdown increased cell apoptosis in ARO cells. Annexin V-PI apoptosis assays were used to examine the cell apoptosis level in ARO-PLKO.1 (A) and LNK-shRNA cells (B and C). [file 12935_2019_1090_MOESM1_ESM.docx]
